# Enhanced photocatalytic, electrochemical and photoelectrochemical properties of TiO_2_ nanotubes arrays modified with Cu, AgCu and Bi nanoparticles obtained via radiolytic reduction

**DOI:** 10.1016/j.apsusc.2016.06.066

**Published:** 2016-11-30

**Authors:** Michał Nischk, Paweł Mazierski, Zhishun Wei, Katarzyna Siuzdak, Natalie Amoin Kouame, Ewa Kowalska, Hynd Remita, Adriana Zaleska-Medynska

**Affiliations:** a Department of Chemical Technology, Faculty of Chemistry, Gdansk University of Technology, 11/12 G. Narutowicza 11/12 St., 80-233 Gdansk, Poland; b Department of Environmental Technology, Faculty of Chemistry, University of Gdansk, 63 Wita Stwosza St., 80-308 Gdansk, Poland; c Institute for Catalysis, Hokkaido University, N21, W10, 001-0021, Sapporo, Japan; d Centre for Plasma and Laser Engineering, The Szewalski Institute of Fluid-Flow Machinery, Polish Academy of Sciences, 14 Fiszera St., 80-231 Gdansk, Poland; e Laboratoire de Chimie Physique, CNRS—UMR 8000,Université Paris-Sud, Université Paris-Saclay, Bâtiment 349, 91405 Orsay, France

**Keywords:** TiO_2_ nanotubes, Metal nanoparticles, Radiolysis, Phenol degradation, Photoelectrochemical performance

## Abstract

TiO_2_ nanotubes arrays (NTs), obtained via electrochemical anodization of Ti foil, were modified with monometallic (Cu, Bi) and bimetallic (AgCu) nanoparticles. Different amounts of metals’ precursors were deposited on the surface of NTs by the spin-coating technique, and the reduction of metals was performed via gamma radiolysis. Surface modification of titania was studied by EDS and XPS analysis. The results show that AgCu nanoparticles exist in a Ag_core_-Cu_shell_ form. Photocatalytic activity was examined under UV irradiation and phenol was used as a model pollutant of water. Over 95% of phenol degradation was achieved after 60 min of irradiation for almost all examined samples, but only slight difference in degradation efficiency (about 3%) between modified and bare NTs was observed. However, the initial phenol degradation rate and TOC removal efficiency was significantly enhanced for the samples modified with 0.31 and 0.63 mol% of Bi as well as for all the samples modified with Cu and AgCu nanoparticles in comparison with bare titania nanotubes. The saturated photocurrent, under the influence of simulated solar light irradiation, for the most active Bi- and AgCu-modified samples, was over two times higher than for pristine NTs. All the examined materials were resistant towards photocorrosion processes that enables their application for long term processes induced by light.

## Highlights

• TiO_2_ nanotubes were modified with Cu, AgCu, Bi nanoparticles via gamma radiolysis.
• Excessive amount of deposited metal decreased photocatalytic activity.
• AgCu-modified samples were more active than Cu-modified (with the same Cu content).
• AgCu nanoparticles exist in a core_(Ag)_-shell_(Cu)_ form.
• Examined photocatalysts were resistant towards photocorrosion processes.

## Graphical abstract

**Figure:**
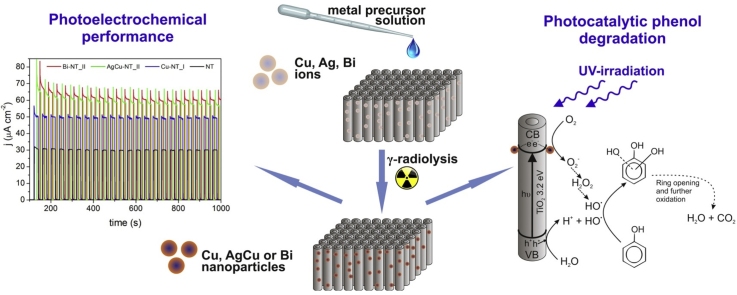

## 1 Introduction

The photocatalysts in the form of ordered arrays of TiO_2_ nanotubes (NTs), electrochemically synthesized directly on titanium surface, attract much attention due to their high specific surface area, enhanced charge carriers transfer, large number of active sites [1], high chemical and mechanical stability [2]. TiO_2_ NTs were utilized for photocatalytic purification of water [3] and gas [4], [5] phases, hydrogen generation [6], [7] and in non-photocatalytic applications such as gas sensors [8], [9], biomedical materials [10], etc.

Various methods such as non-metal (e.g. N [11], B/N [12], S [13]) and transition metal (e.g. Zr [14], Fe [15], [16], Cr [17], [18], Zn [19]) doping were used to enhance the photocatalytic and photoelectrochemical activity of TiO_2_ nanotubes. Another approach to this issue is the modification of nanotubes’ surface with metal (noble and non-noble) nanoparticles [20]. Pd-modified TiO_2_ NTs exhibited enhanced photoactivity in methyl orange degradation process [21], Pt nanoparticles were used to improve photocatalytic (degradation of methyl orange) [22], photoelectrochemical (degradation of galactose) [23] and electrochemical [24] properties of titania nanotubes. Ag-, Au- and Cu-decorated NTs possessed higher photocatalytic activity, comparing to bare NTs, in a process of degradation of “Congo red” [25]. The photocatalytic activity (degradation of methylene blue) of TiO_2_ and WO_3_-TiO_2_ NTs was improved by the deposition of Co [26] or Pt [27] nanoparticles. It has been established that metal nanoparticles present on the surface of TiO_2_ (in the case of difference in their Fermi levels) can act as traps for photoinduced electrons, preventing from fast charge carriers recombination [28], [29], [30]. Moreover, the nanoparticles can induce additional electronic states in the bandgap of TiO_2_, and, as a consequence, the photons with lower energy than inherent band gap of titanium dioxide can cause the generation of electron-hole pairs. Additionally, metal nanoparticles, which often possess catalytic properties, can act as active sites for photocatalytic processes [31], [32], [33]. Also the modification of TiO_2_ with bimetallic nanoparticles attracts much attention, due to large variety of possible combinations (different types and amounts of metals) which allows facilitating the design of materials with desired properties. It was often observed, that bimetallic nanoparticles exhibit synergistic properties compared to their monometallic counterparts [34]. Titania modified with bimetallic nanoparticles such as PdPt [35], AgPt, AuAg [36], AuCu [29], [36], AuPd [32], AgCu [30] NiAu [37] displayed higher catalytic and photocatalytic activity in the comparison with the samples decorated with only one metal.

Among various techniques which were used for deposition of metal nanoparticles on the surface of TiO_2_ nanotubes arrays, photodeposition attracted much attention due to simplicity and mild operating condition of the process [2], [22], [24], [25], [31], [32], [38], [39], [40]. Other deposition techniques were e.g. electrochemical deposition [41], [42], [43], [44], [45], [46], chemical reduction [23], [47], [48], [49], [50], electrophoresis [1], sputtering in vacuum [51]. Another technique, which was used for the synthesis fine metal nanoparticles of controlled size, homogenously distributed on the surface of TiO_2_, is radiolysis [52]. This method was successfully applied to decorate TiO_2_ (in a powder form) with Ag [28], Pt [53], Pd, [54], AuCu [55] and AgCu [30] nanoparticles. Although the radiolytic preparation of metal clusters (such as Pt, Pd, Ru) on the surface of carbon nanotubes was described in many papers [56], [57], [58], [59], to our best knowledge, no reports confirm the use of radiolysis technique to deposit metal nanoparticles on the surface of ordered TiO_2_ nanotubes arrays.

A large number of examples of the use of TiO_2_ nanotubes decorated with monometallic Cu or Ag nanoparticles can be found in the literature [2], [31], [38], [39], [40], [42], [44], [45], [46], [47], [48], [49], [50], [60]. According to previously discussed examples, it is supposed that in the case of modification of TiO_2_ with bimetallic AgCu nanoparticles, a synergistic effect could be observed. However, there are very few studies on modification of titania with bimetallic AgCu nanoparticles. TiO_2_ P25 was co-impregnated with Cu and Ag and subsequently tested in the process of photocatalytic Acid Orange 7 removal [61]. AgCu nanoalloys were also used as sensitizers for metal-cluster-sensitized solar cells [62]. In both cases, the synergistic effect was observed. Recently, Ag@CuO nanoparticles were generated on TiO_2_ by radiolysis [30]. It has been found that the photocatalytic activity of Ag@CuO/P25 is higher under UV light, but lower under visible light compared to the activity of CuO/P25 and Ag/P25 [30]. The application of AgCu nanoparticles for modification of ordered TiO_2_ nanotubes arrays has not yet been reported.

Bismuth is one of the most promising non-noble metals used to enhance photocatalytic activity of TiO_2_. However, in most research, the properties of Bi-doped TiO_2_ photocatalysts (mixed Bi-Ti-oxide phases, Bi_2_O_3_-TiO_2_ composites) obtained via sol-gel [63], [64], [65], [66] and hydrothermal [67] methods were examined. There is also an example of TiO_2_ nanotubes ordered arrays modified with Bi_2_O_3_ using dip coating method, exhibiting high photoelectrocatalytic properties [68] and an example of Bi-doped TiO_2_ NTs (used for energy storage) obtained via direct anodization of Bi-Ti alloy. Only a few reports concern the fabrication of TiO_2_ modified with zero-valent bismuth nanoparticles. However, TiO_2_ P25 modified with zero-valent Bi clusters obtained via radiolytic reduction exhibited enhanced photoactivity (compared with pure TiO_2_), combined with high stability [69]. Although the TiO_2_ nanotubes arrays modified with zero-valent Bi nanoparticles (used as photoelectrochemical sensors for organic compounds detection) was described [70], for the best of our knowledge no reports concerning photocatalytic applications of this kind of arrangement were published.

In this regard, the aim of this work was to investigate the properties of highly ordered TiO_2_ nanotubes arrays decorated with monometallic (Bi, Cu, Ag) and bimetallic (AgCu) nanoparticles obtained using gamma radiolysis technique. The ratio of Cu to Ag in bimetallic nanoparticles was adjusted to the value 3:1 based on preliminary experiments performed with the use of P25 TiO_2_ powder_,_ which is in accordance with the most suitable ratio value mentioned in the literature [62]. The samples were characterized by FE-SEM, STEM, EDS, XRD, XPS techniques. The influence of the amount of metals’ modifiers on electrochemical, photoelectrochemical and photocatalytic (degradation of phenol) performance of modified nanotubes was investigated.

## 2 Experimental

### 2.1 Materials and chemicals

Titanium foil (thickness 0.127 mm, 99.7% purity) purchased from Sigma-Aldrich was used as substrate material. Isopropanol, acetone and methanol (p.a., POCh) were used for cleaning Ti foil surface. NH_4_F (p.a.) and ethylene glycol (99.0%, p.a.) purchased from POCh were the components of electrolyte used for TiO_2_ nanotubes preparation. CuSO_4_·5H_2_O, Ag_2_SO_4_, Bi_2_O_3_ (p.a., FLUKA) were used as precursors of metals used for surface modification of the synthesized titania nanotubes. Isopropanol and ethanol (p.a. Sigma-Aldrich) were used as metal precursors’ solvents. Phenol (≥99%, Sigma-Aldrich) was used as model organic water contaminant. Na_2_SO_4_ (99.5%) used for electrochemical and photoelectrochemical measurements was purchased from POCh Avantor. All the reagents were used without further purification. Deionized water was used as solvent.

### 2.2 Synthesis of ordered TiO_2_ nanotubes layers

**Fig. 1:**
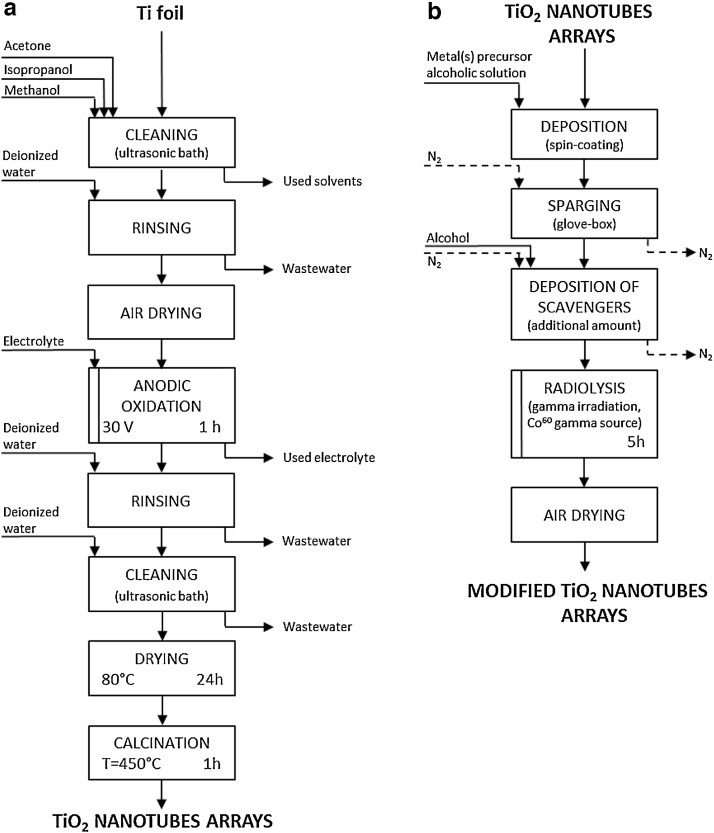
Schematic diagram of preparation procedure of pure TiO_2_ nanotubes (a) and TiO_2_ nanotubes modified with metal nanoparticles (b).

Ordered TiO_2_ nanotubes arrays (NT) were synthesized via simple electrochemical anodization of titanium foil, according to the procedure described in details in our previous paper [5], which is schematically illustrated in Fig. 1a. Ti foil samples (dimensions 2 × 3 cm) were successively ultrasonically cleaned in acetone, isopropanol, methanol, rinsed with water and subsequently dried in air and anodized for 1 h at the applied potential of 30 V in the electrolyte containing ethylene glycol (98%, v/v), water (2%, v/v) and NH_4_F (0.09 M) using 2-electrode system with a cylindrical Pt mesh cathode. Ti foils were immersed in the electrolyte to the level of 2/3 of their length and placed inside the Pt mesh cylinder. Afterwards, the as-synthesized nanotubes were rinsed with water, ultrasonically treated (5 min in water) in order to remove surface precipitates, dried in air (24 h at 80 °C) and calcined for 1 h at 450 °C (heating rate 2 °C min^−1^).

### 2.3 Modification of TiO_2_ NTs with metals via radiolysis technique

Pure TiO_2_ nanotubes were modified with mono- and bimetallic nanoparticles (Cu, Ag, AgCu, Bi) by direct surface adsorption of metals ions or metal precursor particles (Bi) from alcoholic solutions or suspensions followed by radiolytic reduction (Fig. 1b). For monometallic Cu- and Ag-modified NTs, the solutions of CuSO_4_·5H_2_O (10^−3^ M Cu) and Ag_2_SO_4_ (10^−3^ M Ag) in ethanol were prepared. Bimetallic, AgCu-modified samples (Cu:Ag = 3:1) were obtained from irradiation of ethanolic solution containing both CuSO_4_·5H_2_O (10^−3^ M Cu) and Ag_2_SO_4_ (0.33 × 10^−3^ M Ag). The suspension of Bi_2_O_3_ (10^−3^ M Bi) in isopropanol was used for the preparation of Bi-modified nanotubes. The type of the modifier was indicated on the label of the sample (Table 1). In order to prepare metal-modified NTs, a specified amount of the precursor solution was dropped onto the pure nanotubes’ surface using the spin-coating method. The samples to which 3, 7, 14, 21 drops were added (approx. drop volume = 0.018 mL) were denoted as I, II, III, IV respectively (Table 1) with the exception of Ag-NT, where only the sample with 7 drops of precursor’s solution was prepared. It should be noted that only one side of each sample was modified. After precursors’ deposition, the samples were subsequently placed in the glove-box and additional amount of alcohol (which scavenges the oxidizing •OH radicals induced by water radiolysis to lead to alcoholic reducing radicals [69]) was added under N_2_ atmosphere. The samples were then placed in plastic boxes (sealed with parafilm in order to avoid the access of oxygen from air) and exposed to irradiation for 5 h. Metal nanoparticles were synthesized by radiolytic reduction of the metal precursors using a ^60^Co panoramic γ-source located at the Laboratoire de Chimie Physique, in Orsay (dose rate = 3 kGy h^−1^, dose = 15 kGy). After drying in air, the samples were ready for further experiments.

### 2.4 Characterization techniques

The non-modified and meal-modified TiO_2_ nanotubes arrays were characterized by field-emission scanning electron microscopy combined with energy dispersive X-ray spectroscopy (FE-SEM/EDS), scanning transmission electron microscopy combined with energy dispersive X-ray spectroscopy (STEM/EDS), X-ray diffractometry (XRD) and X-ray photoelectron spectroscopy (XPS).

FE-SEM images and EDS spectra were recorded using JEOL JSM-6360LA microscope equipped with JED-2300 energy dispersive X-ray analyzer. The accelerating voltage was 5 kV at working distance 6 mm. Surface elemental analysis was performed in five different areas of the sample in order to determine the average composition.

STEM images and EDS surface mapping were registered using HITACHI HD2000 microscope (accelerating voltage 200 kV, emission current 20 μA). To prepare samples for analysis, the nanotubes were scratched from the substrate (Ti foil) and dispersed in ethanol. The suspension was subsequently dropped on a carbon-covered copper microgrid and dried in vacuum.

The XRD patterns were recorded using Rigaku SmartLab X-ray diffractometer with copper Kα target (40 kV, 30 mA, λ = 1.5404 Å). The scanning range was 2θ = 10–90° at a scan step of 0.01°. Estimated crystal size was calculated according to Sherrer’s formula.

XPS analysis was conducted using Jeol JPC-9010 MC X-ray photoelectron spectrometer with Mg Kα X-ray source. 20 scans were performed for analyzing Ti, O and C content, whereas 50 scans were performed to analyze the presence of metals (Cu, Ag, Bi) used for surface modification of TiO_2_ NTs.

### 2.5 Photocatalytic phenol decomposition measurements

The photocatalytic activity under UV–vis irradiation of non-modified and metal-modified TiO_2_ nanotubes was studied in the process of water purification using phenol (concentration 60 mg L^−1^) as a model pollutant. Photocatalytic activity tests were performed using a simple experimental set-up containing 300 W xenon lamp (Oriel LSH302) equipped with water IR cut-off filter (8 cm length). A quartz cuvette (dimensions 1 × 1 cm) was placed in front of the water filter and acted as a photoreactor. To perform photocatalytic experiment, the photocatalyst sample with the dimensions of 1 × 3 cm (cut from larger sample) and with active part with the dimensions of 1 × 2 cm was placed at the back wall of the cuvette using a paperclip as a holder. Subsequently, the cuvette was filled with 3.5 mL of phenol solution. After 5 min in dark (to ensure the adsorption equilibrium of phenol) the reaction system was irradiated for 60 min. Magnetic stirring and bubbling with oxygen was ensured during the whole period of photocatalytic experiment.

Small aliquots (0.5 mL) of the solution were collected from the reaction system after 20, 40 and 60 min of irradiation in order to analize the phenol degradation by high-performance liquid chromatography (HPLC) and to determine the concentration of organic carbon by total organic carbon (TOC).

The chromatographic system used for HPLC was Agilent 1260 infinity quaternary LC equipped with UV-detector (set at 254.4 nm), the column Adsorbosphere C18 reverse phase (5 μm, l = 150 mm, ID: 4.6 mm; Alltech) combined with an All-Guard cartridge system™ (7.5 × 4.6 mm; Alltech) and the autosampler. The mobile phase (flow rate 1 mL min^−1^) consisted of 75% water and 25% acetonitrile. The peaks areas corresponding to phenol and its main degradation by-products (dihydroxybenzenes) were calculated in direct concentration (mg L^−1^) and in concentration of organic carbon (μmol L^−1^) based on calibration curves.

The TOC measurements were performed using a Shimadzu TOC-LCSH analyzer. Prior to analysis, the samples were diluted in water in order to reach the required sample’s volume. The inorganic carbon present in the samples was removed by acidification and air purging and subsequently TOC was measured by IR after complete catalytic oxidation on Pt catalyst at 680 °C.

### 2.6 Determination of electrochemical and photoelectrochemical properties

The electrochemical and photoelectrochemical activity of pure and modified titania nanotubes was studied using an AutoLab PGStat 302 N potentionstat-galvanostat system (Methrom Autolab) in the standard three-electrode assembly, with titanium foil covered by nanotubes as a working electrode, while Ag/AgCl/0.1 M KCl and Pt mesh as reference and counter electrodes, respectively. The photoelectrochemical cell was equipped with a quartz window and a cooling jacket that keeps the temperature constant at 23 °C (±1 °C) using thermostat (Julabo F-12). Prior to analysis, 0.5 M Na_2_SO_4_ solution of electrolyte was purged with argon gas for about 1 h. Ar-cushion above the electrolyte was applied during the measurements. The transient photocurrent measurements were carried out at + 0.5 V vs. Ag/AgCl/0.1 M KCl bias voltage. A high-pressure 150 W xenon lamp (Osram XBO 150) equipped with AM1.5 filter and the automated light chopper with a period of 20 s was used as a light source. The light intensity was adjusted to 100 mW cm^−2^ (Ophir).

## 3 Results and discussion

### 3.1 Morphology and structure

#### 3.1.1 FE-SEM and STEM analysis combined with EDS

**Fig. 2:**
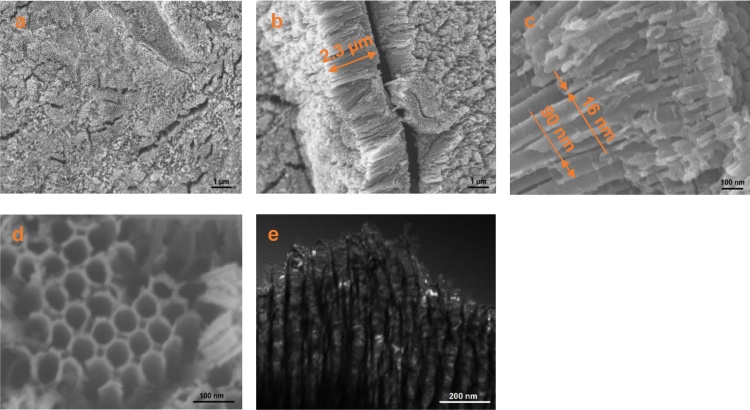
SEM micrographs of pure TiO_2_ nanotubes (a–c) and AgCu-NT_III sample (d); STEM image of AgCu-NT_III sample (e).

FE-SEM images of the non-modified sample (NT) are presented in Fig. 2a-c. The observation confirmed that the surface of Ti substrate was covered with the array of ordered, smooth and elongated nanotubes. However, some scratches and residual precipitates were present on the face of nanotubes, which is in accordance with our previous observations [5]. The FE-SEM images were used to estimate the dimensions of the TiO_2_ nanotubes and to evaluate the total amount of electrochemically synthesized titanium dioxide. The average tube’s length was approximately 2.3 μm and inner diameter and wall thickness were 90 and 16 nm, respectively. The estimated amount of TiO_2_ (one side of the active part of the sample, 2 × 2 cm) was 3.28 mg. This value was used to calculate the content of metals used for surface deposition (Table 1).

In the case of metal-modified samples, FE-SEM and STEM observations (Fig. 2d, e) did not indicate the presence of metal nanoparticles, which is not surprising, taking into account several factors. Radiolysis technique leads to the production of homogenously distributed metal nanoparticles with the size up to several nanometers [52], [55], in the case of Bi not exceeding 1.2 nm [69]. These nanoparticles are probably deposited mainly on the walls’ surface of the nanotubes [52]. Additionally, in the case of copper nanoparticles the small contrast between Cu and TiO_2_ can be a factor that hinders observation [29], [55]. In contrast, another deposition techniques, such as chemical reduction [47], [48], photodeposition [38], [40], [71], electrodeposition [42] led to the formation of larger nanoparticles which were present also on the top surface of nanotubes and could be easily observed using SEM technique.

**Fig. 3:**
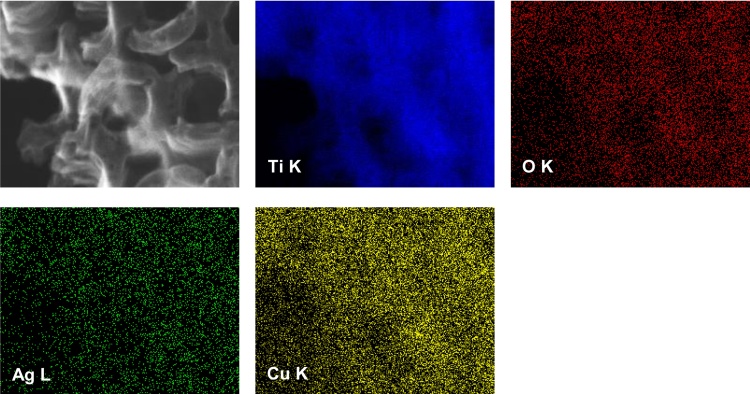
STEM-EDS mapping images of AgCu-NT_III sample.

The presence of the metals used for deposition was confirmed by FE-SEM/EDS analysis (performed for samples with the highest amount of modifiers: Bi-NT_IV, Cu-NT_IV, AgCu-NT_IV). The results of this analysis are presented in Table 1. However, the amount of the deposited metals was much lower than expected, i.e., 0.003, 0.19 and 0.25/0.054 mol% of Bi, Cu and Cu/Ag respectively, due to low precision of EDS analysis for component of low content. The atomic ratios of O to Ti were also much lower than stoichiometric value of two, reaching 0.71, 0.67 and 0.69 for Bi-NT_IV, Cu-NT_IV and AgCu-NT_IV respectively. This is not surprising and has been already observed for other titania samples [72], due to limitation of the EDS method to elements of large atomic number. Low yield of X-ray absorption is noticed for light elements, e.g., oxygen. STEM-EDS mapping analysis performed for sample AgCu-NT_III revealed the homogeneous distribution of metal modifiers on the surface of TiO_2_ nanotubes (Fig. 3).

#### 3.1.2 XPS analysis

The presence of metal nanoparticles as well as chemical composition of surface layer of bare- and metal-modified samples was investigated by X-ray photoelectron spectroscopy (XPS). Six main elements were analyzed in details using narrow scanning, i.e., titanium, oxygen, carbon, bismuth, copper and silver and XPS data is summarized in Table 1 (Bi, Cu and Ag content) and in supplementary materials (Tables S1 and S2). It was found that samples did not differ strongly both in their compositions and chemical state of elements. All the samples possessed carbon on the surface in the range of 18–27 mol%, which could result either from the used electrolyte, or/and adsorbed CO_2_ from the air. It should be mentioned that this amount of carbon is quite low in comparison with titania samples prepared by sol-gel and hydrothermal methods, in which organic precursors of titania (e.g., titanium isopropooxide) are usually used [73]. The atomic ratio of oxygen to titania for pure NT achieved almost the stoichiometric value of 2.0 reaching 1.97. While, for all the modified samples atomic ratios were slightly lower than two (1.61–1.94), probably due to reductive conditions during metal radiolytic deposition on TiO_2_ nanotubes.

The molar content of the deposited metals on the surface layer of NT depended on the kind of the modifiers. It was found that amount of deposited bismuth was lower than expected values (Table 1). It is suggested that either Bi deposition was incomplete, because of too short time used for radiolytic reduction (the same time was applied for all the samples independently on precursor amount) or Bi clusters penetrated and deposited mainly inside the nanotubes. In the case of copper deposition, larger amount of the metal precursor used for deposition resulted in its larger content on the surface of titania nanotubes, with only one exception for Cu-NT_III sample. In the case of deposition of two metals (Ag and Cu), the increase in amount of the metal precursors resulted in proportional increase of their content on the surface layer of NTs. It should be noted that in the case of Cu and AgCu modified samples, the amount of the deposited copper (measured by XPS) was much higher (e.g. 5.6%) than the calculated value (e.g. 0.94%). This phenomenon is not surprising because in the case of surface modification, the modifier is not incorporated into bulk TiO_2_, but accumulates on its surface and, as a consequence, the modifier to titanium ratio increased. This was observed also for titania samples modified with other metals, e.g. Pt [33]. All AgCu-NT samples were prepared using metals’ precursors solution with Cu:Ag ratio equal to 3:1. However, the Cu to Ag ratio determined by XPS was much higher, varying from about 16:1 to 23:1. These results can suggest that bimetallic nanostructures with a core rich in silver and a shell rich in copper are formed. Similar nanoparticles were recently obtained on TiO_2_-P25 by radiolysis [30].

**Fig. 4:**
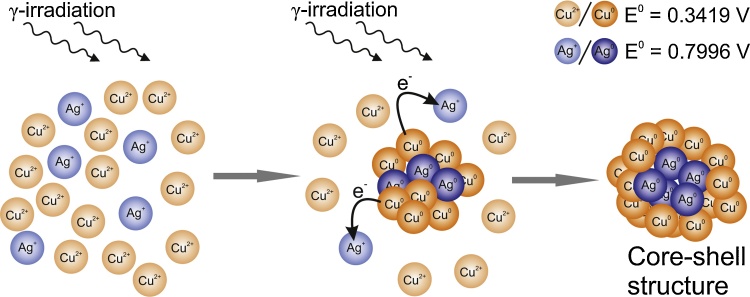
Schematic mechanism of AgCu nanoparticles growth (based on [52,77] and [78]).

According to some theoretical and experimental studies, AgCu clusters tend to form core-shell structures in which copper is located in the core of nanoparticles while silver atoms segregate on the surface and create shell [74], [75], [76]. This is related to such properties of Cu and Ag atoms as relative strength of bonds, surface energies, size and electronegativity [34], [77]. However, here we are out of equilibrium conditions and the radiolytic reduction of metal ions with relatively low gamma dose rate (a few kGy h^−1^) favors the formation of core-shell structures in which the more noble metal is reduced first and form the core of the bimetallic nanoparticle [78]. This is the result of fast electrons’ transfer from less noble metals to more noble ones, privileging their reduction. Hence, the more noble silver atoms are first reduced and create the core of nanoparticle. The core is then covered with the less noble metal (copper) forming the shell (Fig. 4). Copper is sentitive to air. Therefore, the core-shell nanoparticles propably turn into Ag@CuO nanoparticles [30].

**Fig. 5:**
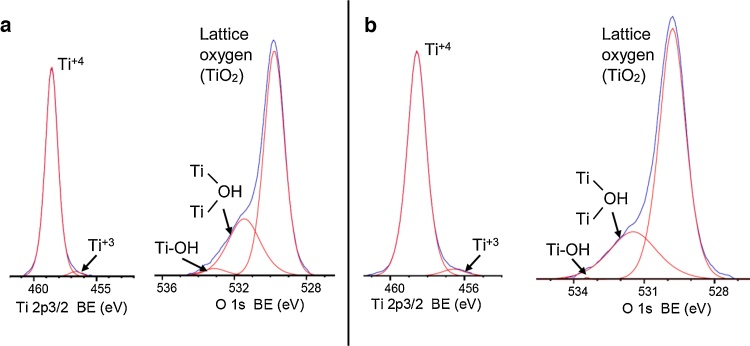
XPS spectra of (left) Ti 2p and (right) O 1s regions for NT (a) and AgCu-NT_III (b) samples.

XPS data after deconvolution of titanium and oxygen peaks are summarized in Table S2 (supplementary materials), and exemplary spectra are shown in Fig. 5. It was found that titanium existed mainly in Ti^+4^ form (>95%). The non-modified TiO_2_ nanotubes contained only ca. 2% of reduced titanium (Ti^+3^). In general, Ti^3+^ formation can be induced by reducing TiO_2_ with a suitable reductant in gas or liquid phase [79]. In the case of TiO_2_ nanotubes obtained with organic electrolytes, oxygen vacancies can be created during calcination step due to high carbon content in as-prepared nanotubes [80]. Modification with metals only slightly increased the content of Ti^+3^, due to reductive conditions during metal depositions, indicating that dose of gamma radiation was well assigned. For two samples with the largest amount of copper and silver (Cu-NT_IV and AgCu-NT_IV) titanium existed only in the oxidized form of +4. Oxygen states varied for all the tested samples. O 1s region could be deconvoluted into two peaks at binding energy of 529.7-529.9 eV and 530.8-531.7 corresponding to lattice oxygen in TiO_2_ and surface oxygen in Ti-OH, respectively. It was found that pure TiO_2_ nanotubes and copper modified Cu-NT_II sample possessed the largest amount of oxygen in the form of surface hydroxyl groups (35.2 and 36.9%, respectively). Interestingly, the samples (Cu-NT_IV and AgCu-NT_IV) possessing titanium in only oxidized form (Ti^+4^) exhibited also the lowest amount of oxygen in the form of hydroxyl group, i.e., the largest amount of oxygen in TiO_2_ form (lattice oxygen) proving the lack of oxygen vacancies (Ti^+3^).

It should be underlined that the positions of titanium peaks did not shift after modification with metals indicating surface modification of titania, but not metal substitution.

#### 3.1.3 XRD analysis

**Fig. 6:**
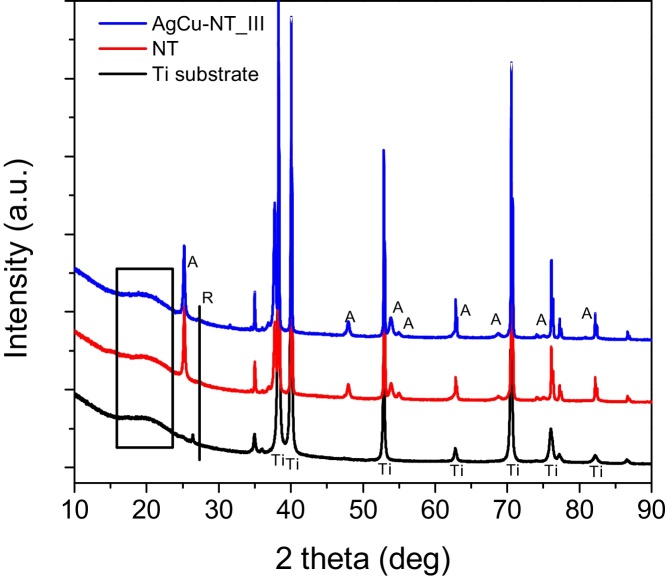
XRD patterns of Ti substrate, pure TiO_2_ nanotubes and AgCu-NT_III sample.

The crystalline composition of non-modified and metal-modified TiO_2_ NTs films was examined by XRD analysis. The exemplary XRD patterns are shown in Fig. 6. Titanium foil consisted mainly of pure titanium with a small amount of crystalline titania, i.e., anatase and rutile in the amount of 5.7 and 7.8 wt.%, respectively. Pure titania nanotubes consisted mainly of amorphous and anatase titania (crystallite size of ca. 35 nm) with a small amount of rutile phase (<5 wt%). It was impossible to determine the precise crystalline composition of the nanotubes, due to very intensive titanium peaks from the support. According to [81], the crystallites of anatase tend to grow along the length and the curvature of the nanotubes rather than across the thickness of the tubes’ walls. As mentioned before, the estimated crystallites’ size has been calculated according to Scherrer’s formula from the broadening of anatase (1 0 1) reflection [82], [79]. This method has been already used to determine the crystallite size of anatase in TiO_2_ nanotubes [83], [84], [85]. The accuracy of the calculations has been estimated to be about 20%. The crystallite size of anatase was in the range of 36–40.4 nm (summarized in Table 1) for all the metal-modified samples, indicating that the kind of modifiers and gamma radiation used for metal reduction/deposition did not influence significantly crystalline properties of titania support. Unfortunately, it was impossible to detect crystallites of deposited metals, possibly due to their very small amount, high level of dispersion and small cluster size, which was consistent with previous studies on titania nanotubes modification with small metal clusters [45], [48], [49], [60], [86].

### 3.2 Photocatalytic activity and mechanism discussion

**Fig. 7:**
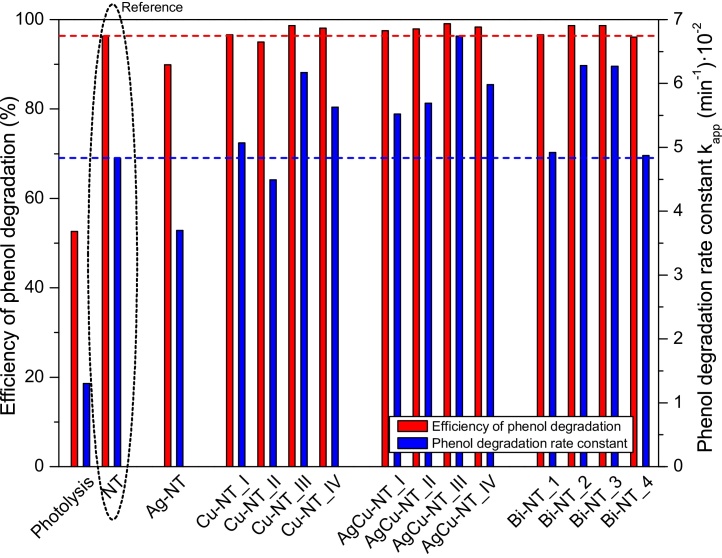
Photocatalytic activity under UV–vis irradiation expressed as efficiency of phenol degradation after 60 min of irradiation and as apparent first order kinetic rate constant. Initial phenol concentration 60 mg L^−1^.

Phenol is a representative of phenolic compounds, which when presents in water, causes severe environmental problems [87] and it is often selected as a model water pollutant in the study of photocatalytic processes [88], [89], [90], [91], [92]. In this regard, it was used to determine the photocatalytic activity of Cu-, AgCu-, and Bi-modified TiO_2_ nanotubes. The results of photocatalytic activity measurements of all the examined samples are presented in Fig. 7. The activity is expressed as the efficiency of phenol degradation (1-c/c_0_) after 60 min of irradiation and as apparent first order kinetic rate constant, calculated from ln(c_0_/c) versus time plot [90]. High efficiency of phenol degradation (90% and more) was observed for all the examined samples. It should be noted that the photolysis had a big influence on the degradation process. About 50% of phenol loss was observed with the absence of photocatalyst. However, it is still much less than in the case of the presence of the less active sample. In this respect, the comparison between the activities of different photocatalysts can be performed.

Among Cu-modified nanotubes, only the samples decorated with larger amount of Cu nanoparticles (Cu-NT_III and IV) exhibited enhanced photocatalytic activity in phenol degradation process compared with bare nanotubes. The efficiency of phenol degradation in the presence of NTs modified with bimetallic (AgCu) nanoparticles was higher in comparison with monometallic samples modified with the same amount of Cu and all the samples were more active than non-modified nanotubes. The sample AgCu-NT_III was the most active. Among Bi-modified NTs, only Bi-NT_II and III samples were more photoactive than bare nanotubes. For comparison, Ag-modified sample was prepared (Ag-NT). The amount of deposited silver was the same as the amount of copper in the most active Cu-modified sample (Cu-NT_III). However, this amount of Ag-modifier caused the decrease of the photoactivity of TiO_2_ nanotubes. This is probably due to exceeding of the optimal Ag content. Wongwisate et al. [93] observed that TiO_2_ modified with relatively small amount of silver (about 0.075 mol%, calculated value) exhibited the highest photoactivity in the initial stage of 4-chlorophenol degradation process. However, silver nanoparticles, when they are present in excessive amount, they can become electron-hole recombination centers and, simultaneously, decrease the photoactivity due to screening effect [61], [93]. In contrast, modification of TiO_2_ with relatively high amount of Cu (0.6 mol%, calculated value) caused the increase in its photocatalytic activity in methyl orange degradation [55]. Moreover, TiO_2_ nanotubes modified with 5.6 mol% Cu (value estimated based on EDX analysis) exhibited higher photoactivity in “Congo red” degradation than pure NTs [25].

**Fig. 8:**
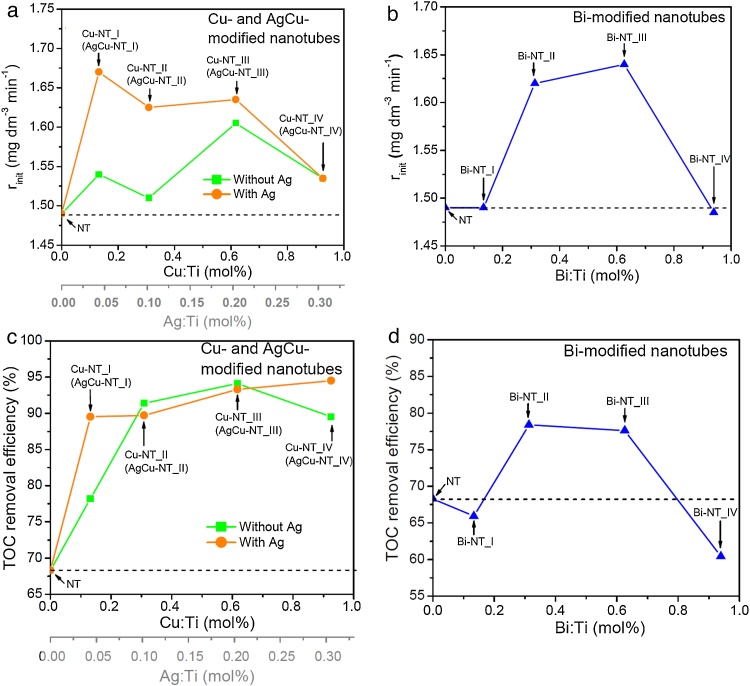
The influence of metal modifier’s amount on photocatalytic activity under UV–vis irradiation expressed as initial phenol degradation rate (a and b) and TOC removal efficiency (c and d).

For more detailed comparison of the prepared samples, another parameters (initial phenol degradation rate and TOC removal efficiency) were considered as more suitable for revealing the differences between the photocatalytic activities of non-modified and metal-modified TiO_2_ nanotubes. Fig. 8a and b show the initial phenol degradation rate in the presence of Cu-, AgCu- and Bi-modified samples, calculated for the first 20 min of irradiation. The initial degradation rate for Ag-NT sample (value not shown in charts) was 1.27 mg dm^−3^ min^−1^, which was lower than for non-modified NTs. The value of this parameter was enhanced for all Cu-modified samples, wherein Cu-NT_III sample was the most active (initial degradation rate about 8% higher than for bare NTs). The presence of silver in Cu nanoparticles caused further increase of the photoactivity, however, the synergistic effect was not observed for the sample decorated with the highest amount of modifier (AgCu-NT_IV). Among all Cu- and AgCu- modified samples, the highest value of initial phenol degradation rate (about 12% higher than for non-modified nanotubes) have been reported for AgCu-NT_I sample and was only slightly lower for AgCu-NT_II and III samples (increase of about 9–9.7% compared to bare nanotubes). Among Bi-modified nanotubes, the highest increase of initial phenol degradation rate was observed for Bi-NT_III sample (about 10%) and only slightly lower for Bi-NT_II sample (about 8.7%). The behavior of the samples modified with the lowest and the highest amount of Bi was similar to non-modified nanotubes.

As it can be seen from Fig. 8c, the TOC removal efficiency after 60 min of irradiation was relatively low (about 68%) in the presence of non-modified TiO_2_ nanotubes. The modification of nanotubes with Cu nanoparticles caused a significant increase in the value of this parameter, reaching over 90% for the samples with higher amount of modifier (Cu-NT_II, III and IV). It should be noted that nanotubes modified with bimetallic, AgCu nanoparticles did not show a meaningful increase of TOC removal efficiency (except from AgCu-NT_I sample) with respect to the samples modified only with copper. However, AgCu-NT_IV sample exhibited the highest TOC removal performance (about 95%) among all Cu- and AgCu-modified nanotubes. Lower effectivity of TOC removal (but still higher than for bare nanotubes) was observed in the case of samples modified with the average amount of Bi nanoparticles (about 78% for samples Bi-NT_II and III), Fig. 8d. The decrease in TOC removal below the level of pure nanotubes was observed for samples with the lowest and the highest Bi-loading (Bi-NT_I and IV respectively).

As can be concluded, for each TiO_2_ modification, an optimal amount of the modifier causes an increase of the photocatalytic activity. In this regard, non-modified nanotubes and metal-modified samples, which exhibited the highest efficiency for phenol degradation, and relatively high values of initial phenol degradation rate and TOC removal efficiency (AgCu-NT_III and Bi-NT_II), were chosen for further mechanism discussion.

**Fig. 9:**
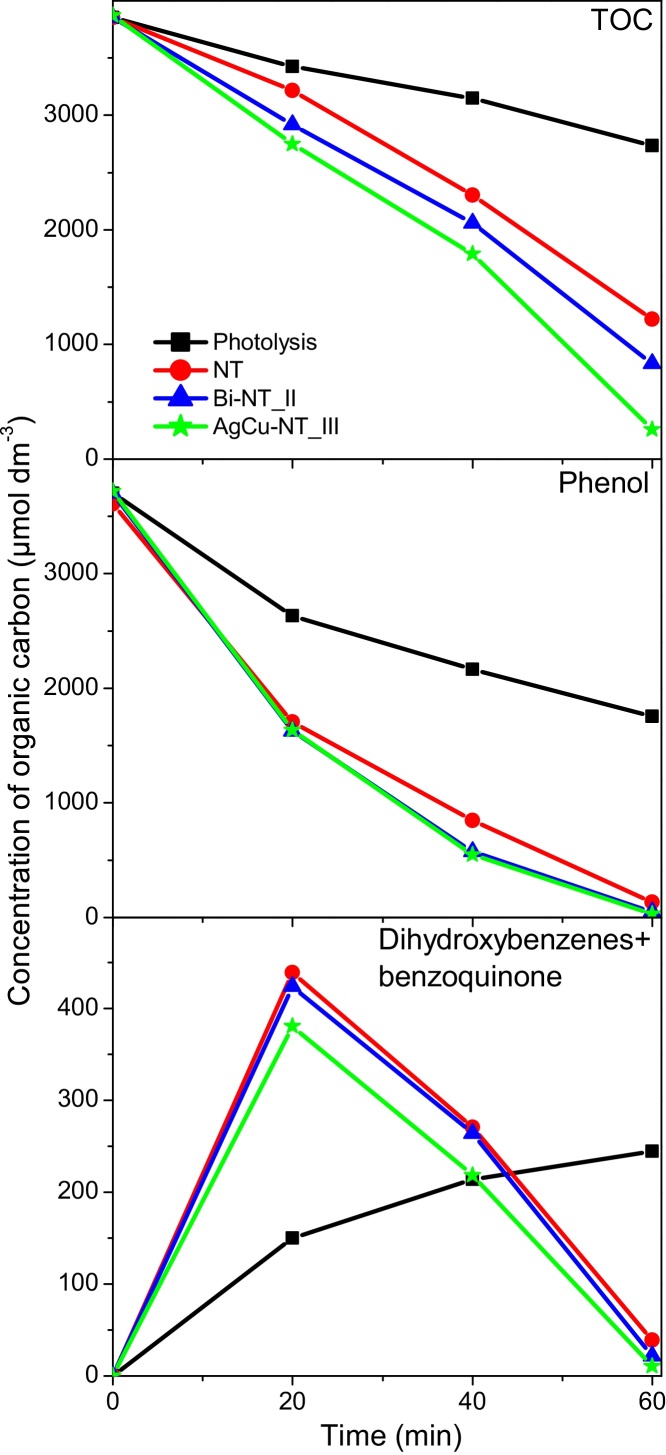
Evolution of TOC, phenol and primary intermediates (expressed as organic carbon content) upon UV–vis irradiation for NT, Bi-NT_II and AgCu-NT_III samples. The results for photolysis are presented for comparison.

**Fig. 10:**
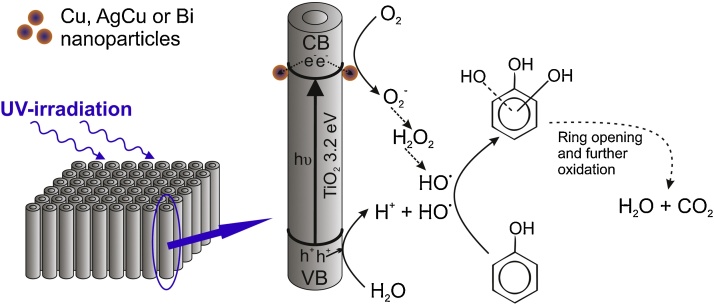
Proposed mechanism of phenol decomposition in the presence of TiO_2_ nanotubes decorated with metal nanoparticles under UV–vis irradiation.

Phenol decomposition in the presence of pure and metal-modified nanotubes was in accordance with a typical scheme of photocatalytic degradation in UV/TiO_2_ system in which the fast initial substrate decay was followed by creation of primary intermediates (hydroquinone, catechol and benzoquinone). After a certain time period, the concentration of primary intermediates reached an optimum and then decreased in parallel with the decrease of the phenol content [54], [82], [94], [95], [96]. As it can be seen from Fig. 9 (and Fig. 8a and b), the initial phenol degradation rate was similar for AgCu-NT_III and Bi-NT_II samples and was higher than for bare nanotubes. The difference in degradation rate increased in the next time period and finally slightly decreased. About 2.5% difference in phenol degradation efficiency between modified- and non-modified nanotubes was observed after 60 min of UV irradiation. The plots of primary intermediates’ concentration vs. time were similar for all the discussed samples, however, after 20 min of irradiation, slightly lower intermediates’ concentration was observed for Bi-modified sample and much lower concentration for AgCu-modified nanotubes. These results suggest that the subsequent photodegradation steps were accelerated by modification of NTs with metal nanoparticles (especially AgCu). This is in agreement with TOC measurements (Figs. 8c and d, 9). It has been observed that the total organic carbon concentration decreased monotonously during the photocatalytic process, however, the degradation rate for pure nanotubes was lower than for Bi- and AgCu-modified samples. After 60 min of irradiation the differences in TOC loss reached 10.1 and 25% (for Bi- and AgCu-modified samples respectively) in comparison with pure nanotubes. Due to the fact that, for all examined samples, the concentrations of phenol and primary intermediates were relatively similar at the end of the process, the higher TOC level can be attributed to the presence of compounds which were formed in subsequent steps of the photocatalytic process. As it was mentioned before, the modification of TiO_2_ with metal nanoparticles can lead to more efficient electron-hole separation and to enhance •OH and O_2_^•−^ radicals formation [30], [32], [33], [53], [54], [55], [93]. O_2_^•−^ radicals can be subsequently transformed, via H_2_O_2_, to •OH radicals which are thought to be the most responsible for photocatalytic degradation of phenol. •OH radicals can be simultaneously generated via direct oxidation of water molecules by photogenerated holes [95]. Thus, higher efficiency of •OH radicals creation can lead to enhanced photoactivity. Guo et al. [92] suggested that the attack of •OH on phenyl ring is the first stage of photocatalytic process which leads to the formation di- and trihydroxybenzenes and, subsequently, to opening of the phenyl ring and forming maleic acid among other intermediate products (Fig. 10).

### 3.3 Electrochemical and photoelectrochemical properties

#### 3.3.1 Linear voltammetry and chronoamperometry

**Fig. 11:**
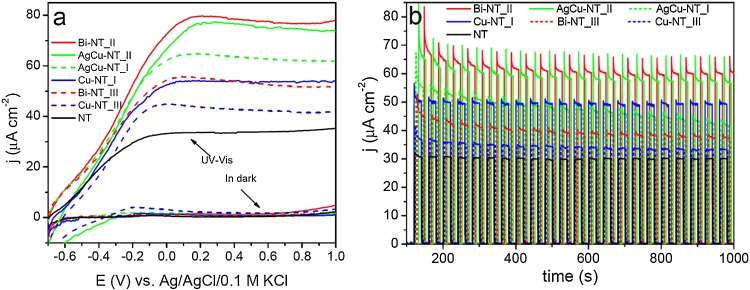
Linear voltamperograms (a) and transient photocurrent response registered at +0.5 V vs. Ag/AgCl/0.1 M KCl (b) of non-modified and selected Cu-, AgCu- and Bi-modified TiO_2_ nanotubes.

The photoelectrochemical behavior of pure and modified titania electrodes exposed to illumination from solar simulator is presented in Fig. 11. The photocurrent densities in all the examined samples increased with the increasing potential applied to the electrode. As shown, TiO_2_ samples with loaded Bi, Cu, and AgCu nanoparticles exhibit improved photoactivity compared to unmodified titania. However, in the case of the two most active TiO_2_ NTs samples: Bi-NT_II and AgCu-NT_II, the saturated photocurrent was over two times higher compared to pristine TiO_2_ NTs. As it was discussed earlier, for Bi-NT_II the highest TOC removal efficiency was observed whereas AgCu-Nt_II sample is characterized with one of the highest initial phenol degradation rate. Thus, enhanced photoelectrochemical activity was reported for the same materials that exhibited improved photocatalytic properties than unmodified titania. In order to investigate the photoelectrode stability, the current was measured at + 0.5 V vs. Ag/AgCl/0.1 M KCl bias voltage for 15 min. Fig. 11b shows the transient photocurrent response for pure and metal modified TiO_2_ nanotube arrays by on-off cycles registered under UV–vis radiation. The run of chronoamperometry curve exhibits the rapid increase and decrease of current when the irradiation was switched on and off, respectively. The dark current densities were negligible for the all tested samples. As it could be seen all the electrode materials were characterized with the resistance towards photocorossion processes that enables their application for long term processes induced by light.

#### 3.3.2 Cyclic voltammetry

**Fig. 12:**
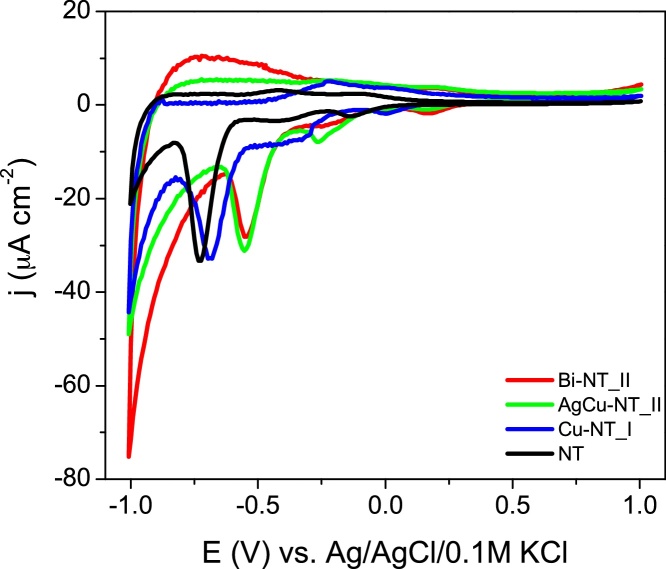
Cyclic voltammograms of non-modified and selected Cu-, AgCu- and Bi-modified TiO_2_ nanotubes. (*v* = 50 mV/s, 0.5 M Na_2_SO_4_).

Cyclic voltammetry (CV) was performed to characterize materials in contact with a deareated electrolyte. Curves registered for pure and Bi, Cu and AgCu modified titania are presented in Fig. 12. The working electrode was polarized from the rest potential in the anodic direction up to 1 V and back up to −1.0 V vs. Ag/AgCl/0.1 M KCl. In general, the CV shape for all the samples is typical for anatase structure of titania NT materials that is characterized with a very low capacitive current in the anodic potential range but in the cathodic range much rich electrochemical activity is observed [97]. In the negative range, two reduction peaks were recorded. According to Bertoluzzi et al. [98] the first one, located at about −0.25 V could be assigned to the filling of narrow deep trap states. On the reverse scan this signal is accompanied with a broader anodic peak that represents relatively slow depopulation of these states [99]. The next signal, recorded at about −0.7 V could be related with alteration in electronic structure of the oxide, *e.g.* incorporation of additional states within the bandgap that change conductivity as well as optical properties [97]. According to Pelouchova et al. [100], this cathodic peak could be ascribed to Ti^4+^ reduction combined with cation intercalation and is related with significant alterations in the material conductivity. On the other hand, for silver modified TiO_2_ NT reduction peak located below −0.5 V was identified as hydrogen evolution [101] despite its generally regarded that this reaction takes place at more negative potentials.

As it could be clearly noticed, CV curves registered for titania samples differ from each other taking into account both positions of cathodic peaks and values of current density. The observed differences result from the presence of metal nanoparticles and increase of Ti^3+^ amount comparing to pristine titania NTs as described above. According to Jiang et al. [1], the observed change in double redox activity in the case of modified TiO_2_ comes from two possible localization of metal nanoparticles: in the internal walls of the nanotubes and at the top surface of the nanoporous structure. Thus, similarly to non-metal doped titania [102], the presence of metal nanoparticles strongly affects the electrochemical activity (e.g. some peaks are shifted or new peaks arises), whereas increased amount of Ti^3+^ can be responsible for the increase of charging current [103].

## 4 Conclusions

In summary, TiO_2_ nanotubes were modified with Cu, AgCu and Bi nanoparticles by using radiolysis technique. EDS and XPS techniques confirmed the presence of metal modifiers, however, the STEM observation did not provide any information about the size and distribution of nanoparticles, probably due to their very small dimensions. Comparing the initial Cu to Ag ratio (3:1) in precursors’ solution with the Cu to Ag ratio (16:1 to 23:1) in AgCu modified samples Ag_core_-Cu_shell_ nanoparticles were obtained. It was observed that surface modification with metal nanoparticles leads to enhanced photocatalytic activity under UV–vis irradiation, and this effect depends on the amount of deposited metal. The efficiency for phenol degradation in the presence of NTs modified with bimetallic (AgCu) nanoparticles was higher in comparison with monometallic samples modified with the same amount of Cu. Among all Cu- and AgCu- modified samples, the highest value of initial phenol degradation rate (about 12% higher than for non-modified nanotubes) is obtained with the bimetallic sample modified with the smallest amount of metals (Cu-0.13 mol%, Ag-0.05 mol%). For Bi-modified samples, the most suitable Bi content was 0.31 and 0.63 mol% in terms of photocatalytic activity. Metal nanoparticles act as traps for excited electrons decreasing the charge carrier recombination rate, and increasing the photocatalytic activity. The photoelectrochemical experiments performed under the influence of simulated solar light irradiation confirmed the enhanced photoactivity of metal-modified nanotubes. The saturated photocurrent for the most active Bi- and AgCu-modified samples, was over two times higher than for pure nanotubes. All the examined materials were resistant towards photocorrosion which enables their application for long term photoinduced processes.

## Figures and Tables

**Table 1:** Samples labeling, content of deposited metal (nominal and measured) and TiO_2_ crystallite size of bare and metal-modified nanotubes.

Sample label	Cu loading^a^ (mol%)			Ag loading^a^ (mol%)			Bi loading^a^ (mol%)			TiO_2_ crystallite size (nm), XRD
	Used for deposition	Analyzed		Used for deposition	Analyzed		Used for deposition	Analyzed		
		XPS	EDS		XPS	EDS		XPS	EDS	
NT	–	–	–	–	–	–	–	–	–	34.9
align="center"										
Ag-NT	–	–	–	0.32	n.a.	n.a.	–	–	–	n.a.
align="center"										
Cu-NT_I	0.13	0.40	n.a.	–	–	–	–	–	–	38.1
Cu-NT_II	0.31	0.83	n.a.	–	–	–	–	–	–	39.5
Cu-NT_III	0.62	0.75	n.a.	–	–	–	–	–	–	40.1
Cu-NT_IV	0.93	4.2	0.19	–	–	–	–	–	–	36.5
align="center"										
AgCu-NT_I	0.13	0.88	n.a.	0.05	0.04	n.a.	–	–	–	36
AgCu-NT_II	0.31	1.8	n.a.	0.11	0.11	n.a.	–	–	–	38.9
AgCu-NT_III	0.63	2.7	n.a.	0.22	0.15	n.a.	–	–	–	36.6
AgCu-NT_IV	0.94	5.6	0.25	0.33	0.24	0.054	–	–	–	36.6
align="center"										
Bi-NT_I	–	–	–	–	–	–	0.13	0.19	n.a.	37.7
Bi-NT_II	–	–	–	–	–	–	0.31	0.07	n.a.	39.9
Bi-NT_III	–	–	–	–	–	–	0.63	0.13	n.a.	40.4
Bi-NT_IV	–	–	–	–	–	–	0.94	0.14	0.003	40.2

a The molar percentage of deposited metal was calculated in reference to TiO_2_.

## References

[1] Jiang Y., Zheng B., Du J., Liu G., Guo Y., Xiao D. (2013). Electrophoresis deposition of Ag nanoparticles on TiO2 nanotube arrays electrode for hydrogen peroxide sensing. Talanta.

[2] Sun L., Li J., Wang C., Li S., Lai Y., Chen H. (2009). Ultrasound aided photochemical synthesis of Ag loaded TiO2 nanotube arrays to enhance photocatalytic activity. J. Hazard. Mater..

[3] Liang H., Li X. (2009). Effects of structure of anodic TiO(2) nanotube arrays on photocatalytic activity for the degradation of 2 3-dichlorophenol in aqueous solution. J. Hazard. Mater..

[4] Kontos A.G., Katsanaki A., Maggos T., Likodimos V., Ghicov A., Kim D. (2010). Photocatalytic degradation of gas pollutants on self-assembled titania nanotubes. Chem. Phys. Lett..

[5] Nischk M., Mazierski P., Gazda M., Zaleska A. (2014). Ordered TiO2 nanotubes: the effect of preparation parameters on the photocatalytic activity in air purification process. Appl. Catal. B Environ..

[6] Zhang Z., Hossain M.F., Takahashi T. (2010). Photoelectrochemical water splitting on highly smooth and ordered TiO2 nanotube arrays for hydrogen generation. Int. J. Hydrogen Energy..

[7] Sun Y., Wang G., Yan K. (2011). TiO2 nanotubes for hydrogen generation by photocatalytic water splitting in a two-compartment photoelectrochemical cell. Int. J. Hydrogen Energy..

[8] Perillo P.M., Rodríguez D.F. (2012). The gas sensing properties at room temperature of TiO2 nanotubes by anodization. Sens. Actuators B Chem..

[9] Şennik E., Çolak Z., Kilinç N., Öztürk Z.Z. (2010). Synthesis of highly-ordered TiO2 nanotubes for a hydrogen sensor. Int. J. Hydrogen Energy.

[10] Brammer K.S., Frandsen C.J., Jin S. (2012). TiO2 nanotubes for bone regeneration. Trends Biotechnol..

[11] Xu J., Ao Y., Chen M., Fu D. (2010). Photoelectrochemical property and photocatalytic activity of N-doped TiO2 nanotube arrays. Appl. Surf. Sci..

[12] Zhou X.S., Peng F., Wang H.J., Yu H., Yang J.A. (2011). Preparation of B,N-codoped nanotube arrays and their enhanced visible light photoelectrochemical performances. Electrochem. Commun..

[13] Momeni M.M., Ghayeb Y., Ghonchegi Z. (2015). Visible light activity of sulfur-doped TiO2 nanostructure photoelectrodes prepared by single-step electrochemical anodizing process. J. Solid State Electrochem..

[14] Liu H., Liu G., Zhou Q. (2009). Preparation and characterization of Zr doped TiO2 nanotube arrays on the titanium sheet and their enhanced photocatalytic activity. J. Solid State Chem..

[15] Wu Q., Ouyang J., Xie K., Sun L., Wang M., Lin C. (2012). Ultrasound-assisted synthesis and visible-light-driven photocatalytic activity of Fe-incorporated TiO2 nanotube array photocatalysts. J. Hazard. Mater..

[16] Momeni M.M., Ghayeb Y. (2015). Fabrication, characterization and photoelectrochemical behavior of Fe–TiO2 nanotubes composite photoanodes for solar water splitting. J. Electroanal. Chem..

[17] Momeni M.M., Ghayeb Y. (2015). Photoelectrochemical water splitting on chromium-doped titanium dioxide nanotube photoanodes prepared by single-step anodizing. J. Alloys Compd..

[18] Momeni M.M., Ghayeb Y. (2016). Fabrication, characterization and photoelectrochemical performance of chromium-sensitized titania nanotubes as efficient photoanodes for solar water splitting. J. Solid State Electrochem..

[19] Momeni M.M., Ghayeb Y. (2015). Visible light-driven photoelectrochemical water splitting on ZnO–TiO2 heterogeneous nanotube photoanodes. J. Appl. Electrochem..

[20] Devi L.G., Kavitha R. (2016). A review on plasmonic metal-TiO2 composite for generation, trapping, storing and dynamic vectorial transfer of photogenerated electrons across the Schottky junction in a photocatalytic system. Appl. Surf. Sci..

[21] Mohapatra S.K., Kondamudi N., Banerjee S., Misra M. (2008). Functionalization of self-organized TiO2 nanotubes with Pd nanoparticles for photocatalytic decomposition of dyes under solar light illumination. Langmuir.

[22] Lv J., Gao H., Wang H., Lu X., Xu G., Wang D. (2015). Applied Surface Science Controlled deposition and enhanced visible light photocatalytic performance of Pt-modified TiO2 nanotube arrays. Appl. Surf. Sci..

[23] Hosseini M.G., Momeni M.M. (2012). Platinum nanoparticle-decorated TiO2 nanotube arrays as new highly active and non-poisoning catalyst for photo-electrochemical oxidation of galactose. Appl. Catal. A Gen..

[24] Tian M., Wu G., Chen A. (2012). Unique electrochemical catalytic behavior of Pt nanoparticles deposited on TiO2 Nanotubes. ACS Catal..

[25] Pearson A., Zheng H., Kalantar-Zadeh K., Bhargava S.K., Bansal V. (2012). Decoration of TiO2 nanotubes with metal nanoparticles using polyoxometalate as a UV-switchable reducing agent for enhanced visible and solar light photocatalysis. Langmuir.

[26] Momeni M.M., Ghayeb Y. (2016). Preparation of cobalt coated TiO2 and WO3–TiO2 nanotube films via photo-assisted deposition with enhanced photocatalytic activity under visible light illumination. Ceram. Int..

[27] Momeni M.M., Ghayeb Y. (2016). Photochemical deposition of platinum on titanium dioxide–tungsten trioxide nanocomposites: an efficient photocatalyst under visible light irradiation. J. Mater. Sci. Mater. Electron..

[28] Grabowska E., Zaleska a., Sorgues S., Kunst M., Etcheberry a., Colbeau-Justin C. (2013). Modification of titanium(IV) dioxide with small silver nanoparticles: application in photocatalysis. J. Phys. Chem. C..

[29] Hai Z., El Kolli D.B., Uribe P., José-Yacaman M., Vigneron J. (2013). Modification of TiO2 by bimetallic Au–Cu nanoparticles for wastewater treatment. J. Mater. Chem. A.

[30] Mendez Medrano M.G., Kowalska E.K., Lehoux A., Herissan A., Ohtani B., Bahena Uribe D. (2016). Surface modification of TiO2 with Ag nanoparticles and CuO nanoclusters for application in photocatalysis. J. Phys. Chem. C.

[31] Gross P.A., Pronkin S.N., Cottineau T., Keller N., Keller V., Savinova E.R. (2012). Effect of deposition of Ag nanoparticles on photoelectrocatalytic activity of vertically aligned TiO2 nanotubes. Catal. Today..

[32] Yu H., Wang X., Sun H., Huo M. (2010). Photocatalytic degradation of malathion in aqueous solution using an Au-Pd-TiO2 nanotube film. J. Hazard. Mater..

[33] Zielińska-Jurek A., Wei Z., Wysocka I., Szweda P., Kowalska E. (2015). The effect of nanoparticles size on photocatalytic and antimicrobial properties of Ag-Pt/TiO2 photocatalysts. Appl. Surf. Sci..

[34] Ferrando R., Jellinek J., Johnston R.L. (2008). Nanoalloys: from theory to applications of alloy clusters and nanoparticles. Chem. Rev..

[35] Rosseler O., Ulhaq-Bouillet C., Bonnefont A., Pronkin S., Savinova E., Louvet A. (2015). Structural and electronic effects in bimetallic PdPt nanoparticles on TiO2 for improved photocatalytic oxidation of CO in the presence of humidity. Appl. Catal. B Environ..

[36] Durán-Álvarez E., Ramírez-Zamora R.M., Zanella R. (2015). Photocatalytic degradation of ciprofloxacin using mono- (Au, Ag and Cu) and bi- (Au-Ag and Au-Cu) metallic nanoparticles supported on TiO2 under UV-C and simulated sunlight. Catal. Today.

[37] Luna A.L., Novoseltceva E., Louarn E., Beaunier P., Kowalska E., Ohtani B. (2016). Synergetic effect of Ni and Au nanoparticles synthesized on titania particles for efficient photocatalytic hydrogen production. Appl. Catal. B Environ..

[38] Momeni M.M. (2015). Applied Surface Science Fabrication of copper decorated tungsten oxide–titanium oxide nanotubes by photochemical deposition technique and their photocatalytic application under visible light. Appl. Surf. Sci..

[39] Ma J., Yang M., Sun Y., Li C., Li Q., Gao F. (2014). Fabrication of Ag/TiO2 nanotube array with enhanced photo-catalytic degradation of aqueous organic pollutant. Phys. E Low-Dimension. Syst. Nanostruct..

[40] Paramasivam I., Macak J.M., Schmuki P. (2008). Photocatalytic activity of TiO2 nanotube layers loaded with Ag and Au nanoparticles. Electrochem. Commun..

[41] Yang L., He D., Cai Q., Grimes C.a. (2007). Fabrication and catalytic properties of Co-Ag-Pt nanoparticle-decorated titania nanotube arrays. J. Phys. Chem. C.

[42] Zhang S., Peng B., Yang S., Wang H., Yu H., Fang Y. (2015). Non-noble metal copper nanoparticles-decorated TiO2 nanotube arrays with plasmon-enhanced photocatalytic hydrogen evolution under visible light. Int. J. Hydrogen Energy..

[43] Li J., Yang L., Luo S., Chen B., Li J., Lin H. (2010). Polycyclic aromatic hydrocarbon detection by electrochemiluminescence generating Ag/TiO 2 nanotubes. Anal. Chem..

[44] Zhang S., Peng F., Wang H., Yu H., Zhang S., Yang J. (2011). Electrodeposition preparation of Ag loaded N-doped TiO2 nanotube arrays with enhanced visible light photocatalytic performance. Catal. Commun..

[45] Xie K., Sun L., Wang C., Lai Y., Wang M., Chen H. (2010). Photoelectrocatalytic properties of Ag nanoparticles loaded TiO2 nanotube arrays prepared by pulse current deposition. Electrochim. Acta..

[46] Luan X., Wang Y. (2013). Preparation and photocatalytic activity of Ag/bamboo-type TiO2 nanotube composite electrodes for methylene blue degradation. Mater. Sci. Semicond. Process..

[47] Wang Q., Qiao J., Xu X., Gao S. (2014). Controlled synthesis of Cu nanoparticles on TiO2 nanotube array photoelectrodes and their photoelectrochemical properties. Mater. Lett..

[48] Hua Z., Dai Z., Bai X., Ye Z., Wang P., Gu H. (2015). Copper nanoparticles sensitized TiO2 nanotube arrays electrode with enhanced photoelectrocatalytic activity for diclofenac degradation. Chem. Eng. J..

[49] Wu F., Hu X., Fan J., Liu E., Sun T., Kang L. (2013). Photocatalytic activity of Ag/TiO2 nanotube arrays enhanced by surface plasmon resonance and application in hydrogen evolution by water splitting. Plasmonics.

[50] Liang Y.Q., Cui Z.D., Zhu S.L., Liu Y., Yang X.J. (2011). Silver nanoparticles supported on TiO2 nanotubes as active catalysts for ethanol oxidation. J. Catal..

[51] Roguska A., Kudelski A., Pisarek M., Opara M., Janik-Czachor M. (2011). Surface-enhanced raman scattering (SERS) activity of Ag, Au and Cu nanoclusters on tiO2-nanotubes/Ti substrate. Appl. Surf. Sci..

[52] Belloni J., Remita H., Spotheim-Maurizot M., Mostafavi M., Douki T., Belloni J. (2008). Metal clusters and nanomaterials. Radiat. Chem. From Basics to Appl. Mater. Life Sci..

[53] Kowalska E., Remita H., Colbeau-Justin C., Hupka J., Belloni J. (2008). Modification of titanium dioxide with platinum ions and clusters: application in photocatalysis. J. Phys. Chem. C.

[54] Tahiri Alaoui O., Herissan A., Le Quoc C., Zekri M.E.M., Sorgues S., Remita H. (2012). Elaboration, charge-carrier lifetimes and activity of Pd-TiO2 photocatalysts obtained by gamma radiolysis. J. Photochem. Photobiol. A Chem..

[55] Hai Z., El Kolli N., Chen J., Remita H. (2014). Radiolytic synthesis of Au–Cu bimetallic nanoparticles supported on TiO2: application in photocatalysis. New J. Chem..

[56] Wang H., Sun X., Ye Y., Qiu S. (2006). Radiation induced synthesis of Pt nanoparticles supported on carbon nanotubes. J. Power Sour..

[57] Rojas J.V., Castano C.H. (2012). Production of palladium nanoparticles supported on multiwalled carbon nanotubes by gamma irradiation. Radiat. Phys. Chem..

[58] Rojas J.V., Toro-Gonzalez M., Molina-Higgins M.C., Castano C.E. (2016). Facile radiolytic synthesis of ruthenium nanoparticles on graphene oxide and carbon nanotubes. Mater. Sci. Eng. B.

[59] Mackiewicz N., Surendran G., Remita H., Keita B., Zhang G., Nadjo L. (2008). Supramolecular self-assembly of amphiphiles on carbon nanotubes: a versatile strategy for the construction of CNT/metal nanohybrids, application to electrocatalysis. J. Am. Chem. Soc..

[60] Wang Y., Li Z., Tian Y., Zhao W., Liu X., Yang J. (2014). Facile method for fabricating silver-doped TiO2 nanotube arrays with enhanced photoelectrochemical property. Mater. Lett..

[61] Behnajady M.A., Eskandarloo H. (2013). Silver and copper co-impregnated onto TiO2-P25 nanoparticles and its photocatalytic activity. Chem. Eng. J..

[62] Shahzad N., Chen F., He L., Li W., Wang H. (2015). Silver–copper nanoalloys-an efficient sensitizer for metal-cluster-sensitized solar cells delivering stable current and high open circuit voltage. J. Power Sour..

[63] Yi W., Yan C., Hamdy M.S., Baltrusaitis J., Mul G. (2014). Effects of bismuth addition and photo-deposition of platinum on (surface) composition, morphology and visible light photocatalytic activity of sol–gel derived TiO2. Appl. Catal. B Environ..

[64] Li L., Huang X., Zhang J., Zhang W., Ma F., Xiao Z. (2015). Multi-layer three-dimensionally ordered bismuth trioxide/titanium dioxide nanocomposite: synthesis and enhanced photocatalytic activity. J. Colloid Interface Sci..

[65] Xu J., Chen M., Fu D. (2011). Study on highly visible light active Bi-doped TiO2 composite hollow sphere. Appl. Surf. Sci..

[66] Solís-Casados D.A., Escobar-Alarcón L., Arrieta-Castañeda A., Haro-Poniatowski E. (2016). Bismuth-titanium oxide nanopowders prepared by sol–gel method for photocatalytic applications. Mater. Chem. Phys..

[67] An’amt M.N., Radiman S., Huang N.M., Yarmo M.A., Ariyanto N.P., Lim H.N. (2010). Sol–gel hydrothermal synthesis of bismuth-TiO2 nanocubes for dye-sensitized solar cell. Ceram. Int..

[68] Zhao X., Liu H., Qu J. (2011). Photoelectrocatalytic degradation of organic contaminants at Bi2O3/TiO2 nanotube array electrode. Appl. Surf. Sci..

[69] Kouamé N.A., Alaoui O.T., Herissan A., Larios E., José-Yacaman M., Etcheberry A. (2015). Visible light-induced photocatalytic activity of modified titanium(IV) oxide with zero-valent bismuth clusters. New J. Chem..

[70] Hu J., Xu G., Wang J., Lv J., Zhang X., Zheng Z. (2014). TiO2 nanotube arrays modified with Bi nanoparticles for enhancing photoelectrochemical oxidation of organics. J. Electrochem. Soc..

[71] Chong X., Zhao B., Li R., Ruan W., Yang X. (2015). Photocatalytic degradation of rhodamine 6G on Ag modified TiO2 nanotubes: surface-enhanced Raman scattering study on catalytic kinetics and substrate recyclability. Colloids Surf. A Physicochem. Eng. Asp..

[72] Reszczyńska J., Grzyb T., Wei Z., Klein M., Kowalska E., Ohtani B. (2016). Photocatalytic activity and luminescence properties of RE3 + −TiO2 nanocrystals prepared by sol–gel and hydrothermal methods. Appl. Catal. B Environ..

[73] Reszczyńska J., Grzyb T., Sobczak J.W., Lisowski W., Gazda M., Ohtani B. (2014). Lanthanide co-doped TiO2: the effect of metal type and amount on surface properties and photocatalytic activity. Appl. Surf. Sci..

[74] Lequien J., Creuze F. (2008). Dynamical equilibrium in nanoalloys. Faraday Discuss..

[75] Lequien F., Creuze J., Berthier F., Braems I., Legrand B. (2008). Superficial segregation, wetting, and dynamical equilibrium in bimetallic clusters: a Monte Carlo study. Phys. Rev. B: Condens. Matter Mater. Phys..

[76] Guisbiers G., Mendoza-Cruz R., Bazán-Díaz L., Velázquez-Salazar J.J., Mendoza-Perez R., Robledo-Torres J.A. (2016). Electrum, the gold–silver alloy, from the bulk scale to the nanoscale: synthesis, properties, and segregation rules. ACS Nano.

[77] Zaleska-Medynska A., Marchelek M., Diak M., Grabowska E. (2016). Bimetallic noble metal nanoparticles: structural, optical, catalytic and photocatalytic properties. Adv. Colloid Interface Sci..

[78] Belloni J., Mostafavi M., Remita H., Marignier J., Delcourt M.-O. (1998). Radiation-induced synthesis of mono- and multi-metallic clusters and nanocolloids. New J. Chem..

[79] Xin X., Xu T., Yin J., Wang L., Wang C. (2015). Management on the location and concentration of Ti3+ in anatase TiO2 for defects-induced visible-light photocatalysis. Appl. Catal. B Environ..

[80] Regonini D., Bowen C.R., Jaroenworaluck A., Stevens R. (2013). A review of growth mechanism, structure and crystallinity of anodized TiO2 nanotubes. Mater. Sci. Eng. R Rep..

[81] Munirathinam B., Pydimukkala H., Ramaswamy N., Neelakantan L. (2015). Influence of crystallite size and surface morphology on electrochemical properties of annealed TiO2 nanotubes. Appl. Surf. Sci..

[82] Ye H., Lu S. (2013). Applied Surface Science Photocatalytic selective oxidation of phenol in suspensions of titanium dioxide with exposed {001} facets. Appl. Surf. Sci..

[83] Kim S.-H., Choi S.-Y. (2015). Fabrication of Cu-coated TiO2 nanotubes and enhanced electrochemical performance of lithium ion batteries. J. Electroanal. Chem..

[84] Nishanthi S.T., Subramanian E., Sundarakannan B., Padiyan D.P. (2015). An insight into the influence of morphology on the photoelectrochemical activity of TiO2 nanotube arrays. Sol. Energy Mater. Sol. Cells.

[85] Cummings F.R., Le Roux L.J., Mathe M.K., Knoesen D. (2010). Structure induced optical properties of anodized TiO2 nanotubes. Mater. Chem. Phys..

[86] Sreekantan S., Zaki S.M., Lai C.W., Tzu T.W. (2014). Copper-incorporated titania nanotubes for effective lead ion removal. Mater. Sci. Semicond. Process..

[87] Chiou C.-H., Wu C.-Y., Juang R.-S. (2008). Influence of operating parameters on photocatalytic degradation of phenol in UV/TiO2 process. Chem. Eng. J..

[88] Turki A., Guillard C., Dappozze F., Ksibi Z., Berhault G., Kochkar H. (2015). Phenol photocatalytic degradation over anisotropic TiO 2 nanomaterials: kinetic study, adsorption isotherms and formal mechanisms. Applied Catal. B Environ..

[89] Wang X., Sø L., Su R., Wendt S., Hald P., Mamakhel A. (2014). The influence of crystallite size and crystallinity of anatase nanoparticles on the photo-degradation of phenol. J. Catal..

[90] Zhang Y., Selvaraj R., Sillanpää M., Kim Y., Tai C. (2014). The influence of operating parameters on heterogeneous photocatalytic mineralization of phenol over BiPO 4. Chem. Eng. J..

[91] Colón G., Sánchez-España J.M., Hidalgo M.C., Navío J.a. (2006). Effect of TiO2 acidic pre-treatment on the photocatalytic properties for phenol degradation. J. Photochem. Photobiol. A Chem..

[92] Guo Z., Ma R., Li G. (2006). Degradation of phenol by nanomaterial TiO2 in wastewater. Chem. Eng. J..

[93] Wongwisate P., Chavadej S., Gulari E., Sreethawong T., Rangsunvigit P. (2011). Effects of monometallic and bimetallic Au–Ag supported on sol–gel TiO2 on photocatalytic degradation of 4-chlorophenol and its intermediates. Desalination.

[94] Moonsiri M., Rangsunvigit P., Chavadej S., Gulari E. (2004). Effects of Pt and Ag on the photocatalytic degradation of 4-chlorophenol and its by-products. Chem. Eng. J..

[95] Rasalingam S., Kibombo H.S., Wu C., Peng R., Baltrusaitis J., Koodali R.T. (2014). Competitive role of structural properties of titania–silica mixed oxides and a mechanistic study of the photocatalytic degradation of phenol. Applied Catal. B Environ..

[96] Sobczyński A., Duczmal Ł., Zmudziński W. (2004). Phenol destruction by photocatalysis on TiO2: an attempt to solve the reaction mechanism. J. Mol. Catal. A Chem..

[97] Macak J.M., Tsuchiya H., Ghicov A., Yasuda K., Hahn R., Bauer S. (2007). TiO2 nanotubes: self-organized electrochemical formation, properties and applications. Curr. Opin. Solid State Mater. Sci..

[98] Bertoluzzi L., Badia-Bou L., Fabregat-Santiago F., Gimenez S., Bisquert J. (2013). Interpretation of cyclic voltammetry measurements of thin semiconductor films for solar fuel applications. J. Phys. Chem. Lett..

[99] Zhang Q., Celorrio V., Bradley K., Eisner F., Cherns D., Yan W. (2014). Density of deep trap states in oriented TiO2 nanotube arrays. J. Phys. Chem. C.

[100] Pelouchova H., Janda P., Weber J., Kavan L. (2004). Charge transfer reductive doping of single crystal TiO2 anatase. J. Electroanal. Chem..

[101] Chen K., Feng X., Hu R., Li Y., Xie K., Li Y. (2013). Effect of Ag nanoparticle size on the photoelectrochemical properties of Ag decorated TiO2 nanotube arrays. J. Alloys Compd..

[102] Siuzdak K., Szkoda M., Sawczak M., Lisowska-Oleksiak A., Karczewski J., Ryl J. (2015). Enhanced photoelectrochemical and photocatalytic performance of iodine-doped titania nanotube arrays. RSC Adv..

[103] Hui W., Chen X., Jing X., Linfeng L., Zhiyong F., Xiaoyuan C. (2013). Enhanced supercapacitance in anodic TiO 2 nanotube films by hydrogen plasma treatment. Nanotechnology.

