# Supplementary material for: Enhanced photocatalytic, electrochemical and photoelectrochemical properties of TiO2 nanotubes arrays modified with Cu, AgCu and Bi nanoparticles obtained via radiolytic reduction
Source: Appl Surf Sci. 2016 Nov 30;387:89–102. doi: 10.1016/j.apsusc.2016.06.066 (PMC5009629; doi:10.1016/j.apsusc.2016.06.066)
Supplement: Supplementary file 1 [file mmc1.docx]

Supporting information for the manuscript

**Enhanced photocatalytic, electrochemical and photoelectrochemical properties of TiO_2_ nanotubes arrays modified with Cu, AgCu and Bi nanoparticles obtained via radiolytic reduction**

Michał Nischk^a,b^, Paweł Mazierski^b^, Zhishun Wei^c,^ Katarzyna Siuzdak^d^, Natalie Amoin Kouame^e^, Ewa Kowalska^c^, Hynd Remita^e^, Adriana Zaleska-Medynska^a,b,*^

## *^a^Department of Chemical Technology, Faculty of Chemistry, Gdansk University of Technology, 11/12 G. Narutowicza 11/12 St., 80-233 Gdansk, Poland*

## *^b^Department of Environmental Technology, Faculty of Chemistry, University of Gdansk, 63 Wita Stwosza St., 80-308 Gdansk, Poland*

## *^c^Institute for Catalysis, Hokkaido University, N21, W10, 001-0021, Sapporo, Japan*

## *^d^Centre for Plasma and Laser Engineering, The Szewalski Institute of Fluid-Flow Machinery, Polish Academy of Sciences, 14 Fiszera St., 80-231 Gdansk, Poland*

## *^e^Laboratoire de Chimie Physique, CNRS – UMR 8000,Université Paris-Sud, Université Paris-Saclay, Bâtiment 349, 91405 Orsay, France*

## * Corresponding author.

## *E-mail address:* adriana.zaleska@ug.edu.pl (A. Zaleska-Medynska)

**Table S1** Chemical composition of bare and metal-modified TiO_2_ NTs based on XPS analysis

| **Sample label** | **Ti 2p3/2 (mol%)** | **O 1s (mol%)** | **O:Ti** | **C 1s (mol%)** | **C:Ti** | **Deposited metal (mol%)** | | | **Deposited metal:Ti** | | |
| --- | --- | --- | --- | --- | --- | --- | --- | --- | --- | --- | --- |
|  |  |  |  |  |  | **Cu 2p3/2** | **Ag 3d5/2** | **Bi 4f7/2** | **Cu:Ti** | **Ag:Ti** | **Bi:Ti** |
| Binding energy (eV) | 458.6 | 529.9 |  | 284.8 |  | 932.0 | 366.8 | 159.0 |  |  |  |
|  | | | | | | | | | | | |
| NT | 24.77 | 48.73 | 1.97 | 26.5 | 1.07 | - | - | - | - | - | - |
|  | | | | | | | | | | | |
| Cu-NT_I | 27.66 | 48.96 | 1.77 | 23.27 | 0.84 | 0.11 | - | - | 0.0040 | - | - |
| Cu-NT_II | 26.41 | 50.03 | 1.89 | 23.34 | 0.88 | 0.22 | - | - | 0.0083 | - | - |
| Cu-NT_III | 26.64 | 49.54 | 1.86 | 23.62 | 0.89 | 0.2 | - | - | 0.0075 | - | - |
| Cu-NT_IV | 26.52 | 48.67 | 1.83 | 23.69 | 0.89 | 1.11 | - | - | 0.042 | - | - |
|  | | | | | | | | | | | |
| AgCu-NT_I | 27.38 | 50.1 | 1.86 | 21.47 | 0.78 | 0.24 | 0.01 | - | 0.0088 | 0.0004 | - |
| AgCu-NT_II | 26.17 | 50.79 | 1.94 | 22.54 | 0.86 | 0.46 | 0.03 | - | 0.018 | 0.0011 | - |
| AgCu-NT_III | 26.47 | 49.65 | 1.87 | 23.14 | 0.87 | 0.71 | 0.04 | - | 0.027 | 0.0015 | - |
| AgCu-NT_IV | 25.41 | 49.51 | 1.94 | 23.6 | 0.93 | 1.42 | 0.06 | - | 0.056 | 0.0024 | - |
|  | | | | | | | | | | | |
| Bi-NT_I | 26.39 | 46.86 | 1.78 | 26.7 | 1.01 | - | - | 0.05 | - | - | 0.0019 |
| Bi-NT_II | 29.11 | 53.20 | 1.83 | 17.68 | 0.61 | - | - | 0.02 | - | - | 0.0007 |
| Bi-NT_III | 31.38 | 50.59 | 1.61 | 17.99 | 0.57 | - | - | 0.04 | - | - | 0.0013 |
| Bi-NT_IV | 28.72 | 48.34 | 1.68 | 22.9 | 0.80 | - | - | 0.04 | - | - | 0.0014 |

**Table S2** Chemical states of elements in bare and metal-modified TiO_2_ NTs based on XPS analysis

| **Sample label** | **Ti 2p3/2 (%)** | | **O 1s (%)** | |
| --- | --- | --- | --- | --- |
|  | **Ti^+4^** | **Ti^+3^** | **-OH** | **TiO_2_** |
| Binding energy (eV) | 458.6 | 456.7 | 529.8 | 531.4 |
|  | | | | |
| NT | 97.82 | 2.18 | 35.2 | 64.8 |
|  | | | | |
| Cu-NT_I | 95.96 | 4.04 | 33.9 | 66.1 |
| Cu-NT_II | 95.57 | 4.43 | 36.9 | 63.1 |
| Cu-NT_III | 96.39 | 3.61 | 31.5 | 68.5 |
| Cu-NT_IV | 100 | 0 | 17.6 | 82.4 |
|  | | | | |
| AgCu-NT_I | 96.64 | 3.36 | 23.2 | 76.8 |
| AgCu-NT_II | 96.03 | 3.97 | 28.3 | 71.7 |
| AgCu-NT_III | 96.44 | 3.56 | 26.0 | 74.0 |
| AgCu-NT_IV | 100 | 0 | 16.5 | 83.5 |
|  | | | | |
| Bi-NT_I | 97.47 | 2.53 | 27.6 | 72.4 |
| Bi-NT_II | 94.89 | 5.11 | 23.5 | 76.4 |
| Bi-NT_III | 96.43 | 3.57 | 18.2 | 81.8 |
| Bi-NT_IV | 95.69 | 4.31 | 24.6 | 75.4 |
